# Complete genome sequence of *Desulfobulbus propionicus* type strain (1pr3^T^)

**DOI:** 10.4056/sigs.1613929

**Published:** 2011-02-20

**Authors:** Ioanna Pagani, Alla Lapidus, Matt Nolan, Susan Lucas, Nancy Hammon, Shweta Deshpande, Jan-Fang Cheng, Olga Chertkov, Karen Davenport, Roxane Tapia, Cliff Han, Lynne Goodwin, Sam Pitluck, Konstantinos Liolios, Konstantinos Mavromatis, Natalia Ivanova, Natalia Mikhailova, Amrita Pati, Amy Chen, Krishna Palaniappan, Miriam Land, Loren Hauser, Yun-Juan Chang, Cynthia D. Jeffries, John C. Detter, Evelyne Brambilla, K. Palani Kannan, Olivier D. Ngatchou Djao, Manfred Rohde, Rüdiger Pukall, Stefan Spring, Markus Göker, Johannes Sikorski, Tanja Woyke, James Bristow, Jonathan A. Eisen, Victor Markowitz, Philip Hugenholtz, Nikos C. Kyrpides, Hans-Peter Klenk

**Affiliations:** 1 DOE Joint Genome Institute, Walnut Creek, California, USA; 2 Los Alamos National Laboratory, Bioscience Division, Los Alamos, New Mexico, USA; 3 Biological Data Management and Technology Center, Lawrence Berkeley National Laboratory, Berkeley, California, USA; 4 Oak Ridge National Laboratory, Oak Ridge, Tennessee, USA; 5 DSMZ - German Collection of Microorganisms and Cell Cultures GmbH, Braunschweig, Germany; 6 HZI – Helmholtz Centre for Infection Research, Braunschweig, Germany; 7 University of California Davis Genome Center, Davis, California, USA; 8 Australian Centre for Ecogenomics, School of Chemistry and Molecular Biosciences The University of Queensland, Brisbane, Australia

**Keywords:** anaerobic, non-motile, Gram-negative, chemoorganotroph, ellipsoidal to lemon-shaped, non spore-forming, mesophilic, *Desulfobulbaceae*, GEBA

## Abstract

*Desulfobulbus propionicus* Widdel 1981 is the type species of the genus *Desulfobulbus*, which belongs to the family *Desulfobulbaceae*. The species is of interest because of its great implication in the sulfur cycle in aquatic sediments, its large substrate spectrum and a broad versatility in using various fermentation pathways. The species was the first example of a pure culture known to disproportionate elemental sulfur to sulfate and sulfide. This is the first completed genome sequence of a member of the genus *Desulfobulbus* and the third published genome sequence from a member of the family *Desulfobulbaceae*. The 3,851,869 bp long genome with its 3,351 protein-coding and 57 RNA genes is a part of the *** G****enomic* *** E****ncyclopedia of* *** B****acteria and* *** A****rchaea * project.

## Introduction

Strain 1pr3^T^ "Lindhorst" (= DSM 2032 = ATCC 33891 = VKM B-1956) is the type strain of the species *Desulfobulbus propionicus*, which is the type species of the genus *Desulfobulbus* [1,2]. The genus currently consists of five validly published named species [3]. The genus name is derived from the Neo-Latin word 'desulfo-' meaning 'desulfurizing' and the Latin word 'bulbus' meaning 'a bulb or an onion', yielding the 'onion-shaped sulfate reducer' [2]. The species epithet is derived from the Neo-Latin word 'acidum propionicum' and the Latin suffix '-icus' in the sense of 'pertaining to'; 'propionicus' = 'pertaining to propionic acid' [2]. Strain 1pr3^T^ "Lindhorst" was isolated by Fritz Widdel in 1982 from anaerobic mud of a village ditch in Lindhorst near Hannover [4]. Other strains have been isolated from anaerobic mud in a forest pond near Hannover and from a mud flat of the Jadebusen (North Sea) [4], from an anaerobic intertidal sediment in the Ems-Dollard estuary (Netherlands) [5], and from a sulfate-reducing fluidized bed reactor inoculated with mine sediments and granular sludge [6]. Several studies have been carried out on the metabolic pathways of the strain 1pr3^T^ [4,7,8]. Here we present a summary classification and a set of features for *D. propionicus* strain 1pr3^T^, together with the description of the complete genomic sequencing and annotation.

## Classification and features

A representative genomic 16S rRNA sequence of strain 1pr3^T^ was compared using NCBI BLAST under default settings (e.g., considering only the high-scoring segment pairs (HSPs) from the best 250 hits) with the most recent release of the Greengenes database [9] and the relative frequencies, weighted by BLAST scores, of taxa and keywords (reduced to their stem [10]) were determined. The four most frequent genera were *Desulfobulbus* (76.1%), *Desulfurivibrio* (11.9%), *Desulforhopalus* (8.1%) and *Desulfobacterium* (3.9%) (19 hits in total). Regarding the eleven hits to sequences from members of the species, the average identity within HSPs was 95.1%, whereas the average coverage by HSPs was 94.7%. Regarding the nine hits to sequences from other members of the genus, the average identity within HSPs was 94.9%, whereas the average coverage by HSPs was 94.9%. Among all other species, the one yielding the highest score was *Desulfobulbus elongatus*, which corresponded to an identity of 96.9% and an HSP coverage of 93.8%. The highest-scoring environmental sequence was FJ517134 (''semiarid 'Tablas de Daimiel National Park' wetland (Central Spain) unraveled water clone TDNP Wbc97 92 1 234'), which showed an identity of 97.8% and a HSP coverage of 98.3%. The five most frequent keywords within the labels of environmental samples which yielded hits were 'sediment' (8.4%), 'marin' (2.9%), 'microbi' (2.5%), 'sea' (1.7%) and 'seep' (1.7%) (231 hits in total). These keywords are in line with habitats from which the cultivated strains of *D. propionicus* were isolated. Environmental samples which resulted in hits of a higher score than the highest scoring species were not found.

**Figure 1:**
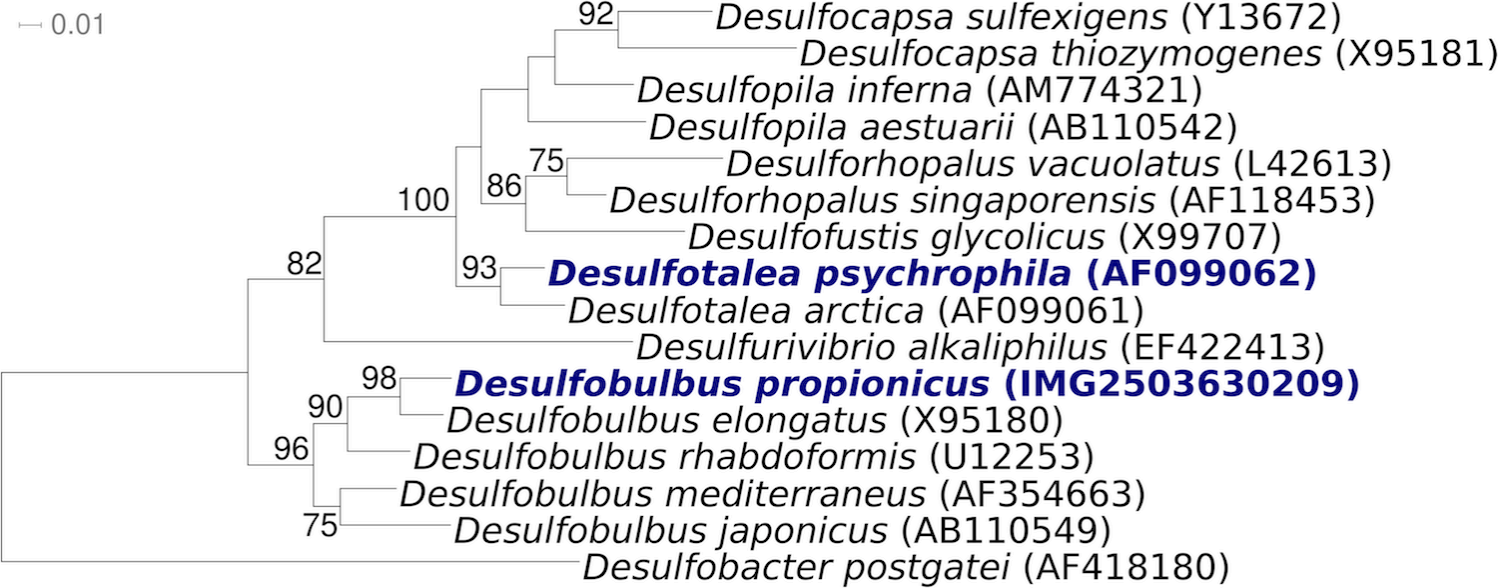
Phylogenetic tree highlighting the position of *D. propionicus* relative to the other type strains within the family *Desulfobulbaceae*. The tree was inferred from 1,425 aligned characters [11,12] of the 16S rRNA gene sequence under the maximum likelihood criterion [13] and rooted in accordance with the current taxonomy. The branches are scaled in terms of the expected number of substitutions per site. Numbers above branches are support values from 200 bootstrap replicates [14] if larger than 60%. Lineages with type strain genome sequencing projects registered in GOLD [15] are shown in blue, published genomes [16] in bold.

Figure 1 shows the phylogenetic neighborhood of *D. propionicus* in a 16S rRNA based tree. The sequences of the two 16S rRNA gene copies in the genome do not differ from each other, and differ by two nucleotides from the previously published 16S rRNA sequence (AY548789).

**Figure 2:**
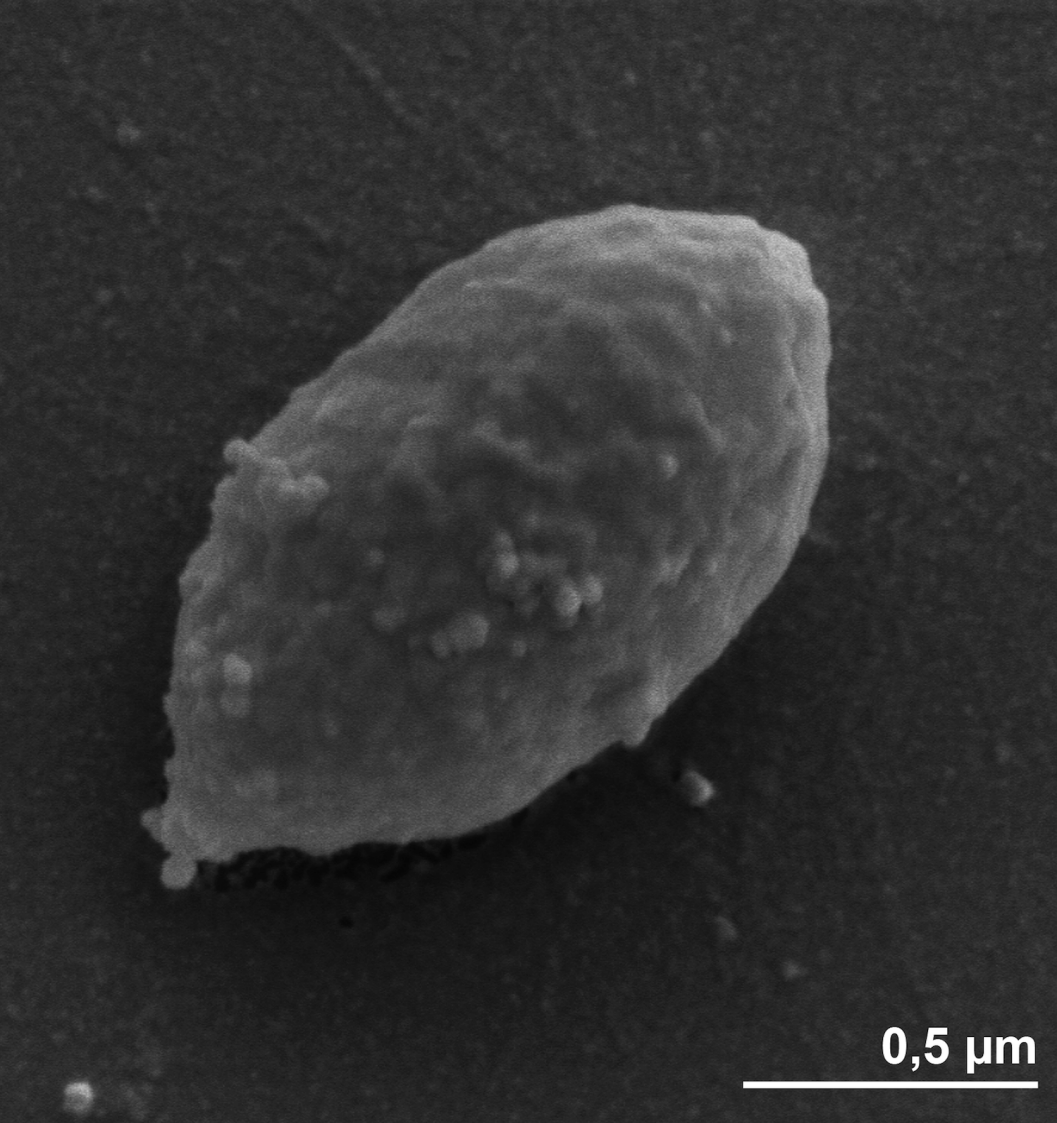
Scanning electron micrograph of *D. propionicus* 1pr3^T^

The cells of *D. propionicus* are ellipsoidal to lemon-shaped (1-1.3 by 1.8-2 µm) (Figure 2). *D. propionicus* is a Gram-negative and non- sporulating bacterium (Table 1) that produces fimbriae [4]. The temperature range for growth is between 10ºC and 43ºC, with an optimum at 39ºC [4]. The pH range for growth is between 6.0 and 8.6, with an optimum at pH 7.1-7.5 [4]. Strain 1pr3^T^ is described to be nonmotile, with no flagellum detected by electron microscopy [4], although the genome sequence suggests it to be comprehensively equipped with the genes required for flagellar assembly (see below). The closely related strains 2pr4 and 3pr10 were motile by a single polar flagellum [4], suggesting either a recent mutational loss of flagellar motility in strain 1pr3^T^, or a failure to express the genes under the conditions of growth. *D. propionicus* was initially described to be a strictly anaerobic chemoorganotroph [4]. Further studies a decade later indicated that this organism was able to grow in the presence of oxygen while oxidizing sulfide, elemental sulfur, sulfite and polysulfide to sulfate [27], where mainly thiosulfate was formed from elemental sulfur [27,28]. *D. propionicus* is the first example of a pure culture known to disproportionate elemental sulfur to sulfate and sulfide [7]. But growth of *D. propionicus* with elemental sulfur as the electron donor and Fe(III) as a sulfide sink and/or electron acceptor was very slow [7]. It ferments three moles of pyruvate to two moles acetate and one mole of propionate stoichiometrically *via* the methylmalonyl-CoA pathway [8]. Strain 1pr3^T^ was also found to reduce iron to sustain growth [7]. Fe(III) greatly stimulated sulfate production, and *D. propionicus* produced as much sulfate in the absence of Mn(IV) or Fe(III) as it did with Mn(IV) [7]. In the absence of sulfate, ethanol is fermented to propionate and acetate in a molar ratio of 2:1 [24], while *i-*propanol is produced during the fermentation of ethanol [24]. In the presence of H_2_ and CO_2_, ethanol is quantitatively converted to propionate [24]. H_2_- plus sulfate-grown cells of the strain 1pr3^T^ were able to oxidize 1-propanol and 1-butanol to propionate and butyrate respectively with the concomitant reduction of acetate plus CO_2_ to propionate [24]. Growth on H_2_ required acetate as a carbon source in the presence of CO_2_ [4]. Strain 1pr3^T^ is also able to grow mixotrophically on H_2_ in the presence of an organic compound [24]. When the amounts of sulfate and ethanol are limiting, *D. propionicus* competes successfully with *Desulfobacter postgatei*, another sulfate reducer [29]. Propionate, lactate, ethanol and propanol were used as electron donors and carbon sources [4]. Together with pyruvate, they are oxidized to acetate as an end-product [4]. Butyrate may be used in a few cases [4]. Sulfide oxidation in *D. propionicus* is biphasic, proceeding *via* oxidation to elemental sulfur, followed by sulfur disproportionation to sulfide and sulfate [7,27,30]. However, the uncoupler tetrachlorosalicylanilide (TCS) and the electron transport inhibitor myxothiazol inhibited sulfide oxidation to sulfate and caused accumulation of sulfur [30]. But in the presence of the electron transport inhibitor 2-*n*-heptyl-4-hydroxyquinoline-*N*-oxide (HQNO), sulfite and thiosulfate were formed [30]. When grown on lactate or pyruvate, the strain 1pr3^T^ is able to grow without an external electron acceptor and formed propionate and acetate as fermentation products [4,31]. For this purpose, the substrates are fermented via the methylmalonyl-CoA pathway [31]. In the cells of *D. propionicus*, the activities of methylmalonyl-CoA: pyruvate transcarboxylase, a key enzyme of methylmalonyl-CoA pathway, as well as the other enzymes (pyruvate dehydrogenase, succinate dehydrogenase and malate dehydrogenase) involved in the pathway were detected [31]. *D. propionicus* can convert not only pyruvate but also alcohols via methylmalonyl-CoA pathway in the absence of sulfate [24,32,33]. Inorganic pyrophosphatase was present in strain 1pr3^T^ at high levels of activity, but the enzyme was Mg^2+^-dependent and stimulated by Na_2_S_2_O_4_ [34]. However, isocitrate lyase and pyrophosphate-dependent acetate kinase were not detected [34]. Sulfate, sulfite and thiosulfate serve as electron acceptors and are reduced to H_2_S, but not elemental sulfur, malate, fumarate [4]. Nitrate also served as electron acceptor and was reduced to ammonia [4,27]. Acetate, valerate, higher fatty acids, succinate, fumarate, malate, sugars are not utilized [4]. Strain 1pr3^T^ requires 4-aminobenzoic acid as growth factor [4,6]. Cell membrane and cytoplasmic fraction contain b- and c-type cytochromes [4].

**Table 1:** Classification and general features of *D. propionicus* 1pr3^T^ according to the MIGS recommendations [17].

MIGS ID	Property	Term	Evidence code
	Current classification	Domain *Bacteria*	TAS [18]
		Phylum *Proteobacteria*	TAS [19]
		Class *Deltaproteobacteria*	TAS [20,21]
		Order *Desulfobacterales*	TAS [20,22]
		Family *Desulfobulbaceae*	TAS [20,23]
		Genus *Desulfobulbus*	TAS [1,2]
		Species *Desulfobulbus propionicus*	TAS [1,2]
		Type strain 1pr3	TAS [4]
	Gram stain	negative	TAS [4]
	Cell shape	ellipsoidal to lemon-shaped	TAS [4]
	Motility	non-motile	TAS [4]
	Sporulation	none	TAS [4]
	Temperature range	10°C-43°C	TAS [4]
	Optimum temperature	39°C	TAS [4]
	Salinity	not reported	NAS
MIGS-22	Oxygen requirement	anaerobic	TAS [4]
	Carbon source	propionate, lactate, ethanol, propanol, pyruvate	TAS [4,6]
	Energy source	chemoorganotroph	TAS [4]
MIGS-6	Habitat	anaerobic freshwater sediments	TAS [24]
MIGS-15	Biotic relationship	not reported	NAS
MIGS-14	Pathogenicity	not reported	NAS
	Biosafety level	1	TAS [25]
	Isolation	anaerobic mud	TAS [4]
MIGS-4	Geographic location	Lindhort near Hannover, Germany	TAS [4]
MIGS-5	Sample collection time	1980 or before	NAS
MIGS-4.1	Latitude	52.38	NAS
MIGS-4.2	Longitude	9.82	NAS
MIGS-4.3	Depth	not reported	NAS
MIGS-4.4	Altitude	not reported	NAS

Evidence codes - IDA: Inferred from Direct Assay (first time in publication); TAS: Traceable Author Statement (i.e., a direct report exists in the literature); NAS: Non-traceable Author Statement (i.e., not directly observed for the living, isolated sample, but based on a generally accepted property for the species, or anecdotal evidence). These evidence codes are from of the Gene Ontology project [26]. If the evidence code is IDA, then the property was directly observed by one of the authors or an expert mentioned in the acknowledgements.

### Chemotaxonomy

Odd-chain fatty acids predominated in the fatty acid profile of the strain 1pr3^T^ (77% of the total fatty acids *vs.* 23% for the even-chain fatty acids) [35,36], reflecting the use of propionate as a chain initiator for fatty acid biosynthesis [35]. The major fatty acids, when grown on propionate, were found to be C_17:1ω6_ (51.5%), C_15:0_ (28.3%), C_16:0_ (6.9%), C_14:0_ (5.2%), C_18:0_ (3.1%), C_15:1 ω6_ and C_16:1 ω5_, (2.4% each) and C_18:1 ω7_ (2.1%). The minor fatty acids were C_17:0_ (0.6% of the total fatty acids), C_16:1 ω7_ (0.9%)_,_ C_18:1 ω9_ and C_15:1Δ7_ (1.0% each), C_12:0_ (1.3%), C_17:1 ω8_ (1.6%) and C_13:0_ (1.7%) [36].

## Genome sequencing and annotation

### Genome project history

This organism was selected for sequencing on the basis of its phylogenetic position [37], and is part of the *** G****enomic* *** E****ncyclopedia of* *** B****acteria and* *** A****rchaea * project [38]. The genome project is deposited in the Genomes OnLine Database [15] and the complete genome sequence is deposited in GenBank. Sequencing, finishing and annotation were performed by the DOE Joint Genome Institute (JGI). A summary of the project information is shown in Table 2.

**Table 2:** Genome sequencing project information

**MIGS ID**	**Property**	**Term**
MIGS-31	Finishing quality	Finished
MIGS-28	Libraries used	Three genomic libraries:one 454 pyrosequence standard library, one 454 PE library (12 kb insert size), one Illumina library
MIGS-29	Sequencing platforms	Illumina GAii, 454 GS FLX Titanium
MIGS-31.2	Sequencing coverage	109.7 × Illumina; 37.9 × pyrosequence
MIGS-30	Assemblers	Newbler version 2.0.00.20- PostRelease-11-05-2008-gcc-3.4.6, Velvet, phrap
MIGS-32	Gene calling method	Prodigal 1.4, GenePRIMP
	INSDC ID	CP002364
	Genbank Date of Release	January 28, 2011
	GOLD ID	Gc01599
	NCBI project ID	32577
	Database: IMG-GEBA	2503538026
MIGS-13	Source material identifier	DSM 2032
	Project relevance	Tree of Life, GEBA

### Growth conditions and DNA isolation

*D. propionicus* 1pr3^T^, DSM 2032, was grown anaerobically in DSMZ medium 194 (*Desulfobulbus* medium) [39] at 37°C. DNA was isolated from 0.5-1 g of cell paste using MasterPure Gram-positive DNA purification kit (Epicentre MGP04100) following the standard protocol as recommended by the manufacturer, with modification st/LALM for cell lysis as described in Wu *et al*. [38]. DNA is available through the DNA Bank Network [40,41].

### Genome sequencing and assembly

The genome was sequenced using a combination of Illumina and 454 sequencing platforms. All general aspects of library construction and sequencing can be found at the JGI website [42]. Pyrosequencing reads were assembled using the Newbler assembler version 2.0.00.20-PostRelease-11-05-2008-gcc-3.4.6 (Roche). The initial Newbler assembly consisting of 35 contigs in two scaffolds was converted into a phrap [43] assembly by making fake reads from the consensus, to collect the read pairs in the 454 paired end library. Illumina GAii sequencing data (327Mb) was assembled with Velvet [44] and the consensus sequences were shredded into 1.5 kb overlapped fake reads and assembled together with the 454 data. The 454 draft assembly was based on 145.0 Mb 454 draft data and all of the 454 paired end data. Newbler parameters are -consed -a 50 -l 350 -g -m -ml 20. The Phred/Phrap/Consed software package [43] was used for sequence assembly and quality assessment in the subsequent finishing process. After the shotgun stage, reads were assembled with parallel phrap (High Performance Software, LLC). Possible mis-assemblies were corrected with gapResolution [42], Dupfinisher [45], or sequencing cloned bridging PCR fragments with subcloning or transposon bombing (Epicentre Biotechnologies, Madison, WI). Gaps between contigs were closed by editing in Consed, by PCR and by Bubble PCR primer walks (J.-F.Chang, unpublished). A total of 563 additional reactions and five shatter libraries were necessary to close gaps and to raise the quality of the finished sequence. Illumina reads were also used to correct potential base errors and increase consensus quality using a software Polisher developed at JGI [46]. The error rate of the completed genome sequence is less than 1 in 100,000. Together, the combination of the Illumina and 454 sequencing platforms provided 147.6 × coverage of the genome. The final assembly contained 475,513 pyrosequence and 11,740,513 Illumina reads.

### Genome annotation

Genes were identified using Prodigal [47] as part of the Oak Ridge National Laboratory genome annotation pipeline, followed by a round of manual curation using the JGI GenePRIMP pipeline [48]. The predicted CDSs were translated and used to search the National Center for Biotechnology Information (NCBI) nonredundant database, UniProt, TIGR-Fam, Pfam, PRIAM, KEGG, COG, and InterPro databases. Additional gene prediction analysis and functional annotation was performed within the Integrated Microbial Genomes - Expert Review (IMG-ER) platform [49].

## Genome properties

**Figure 3:**
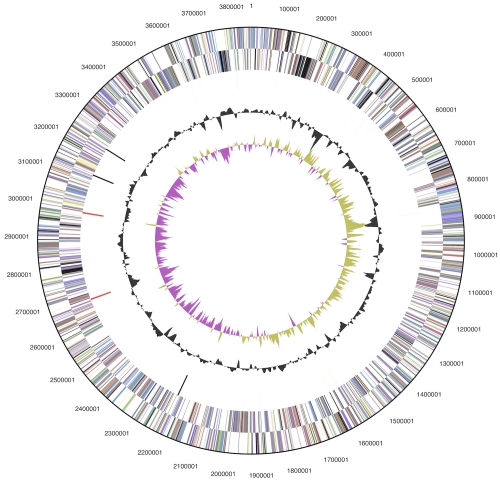
Graphical circular map of the chromosome. From outside to the center: Genes on forward strand (color by COG categories), Genes on reverse strand (color by COG categories), RNA genes (tRNAs green, rRNAs red, other RNAs black), GC content, GC skew.

The genome consists of a 3,851,869 bp long chromosome with a GC content of 58.9% (Table 3 and Figure 3). Of the 3,408 genes predicted, 3,351 were protein-coding genes, and 57 RNAs; 68 pseudogenes were also identified. The majority of the protein-coding genes (70.5%) were assigned with a putative function while the remaining ones were annotated as hypothetical proteins. The distribution of genes into COGs functional categories is presented in Table 4.

**Table 3:** Genome Statistics

**Attribute**	Value	% of Total
Genome size (bp)	3,851,869	100.00%
DNA coding region (bp)	3,410,010	88.53%
DNA G+C content (bp)	2,269,813	58.93%
Number of replicons	1	
Extrachromosomal elements	0	
Total genes	3,408	100.00%
RNA genes	57	1.67%
rRNA operons	2	
Protein-coding genes	3,351	98.33%
Pseudo genes	68	2.00%
Genes with function prediction	2,402	70.48%
Genes in paralog clusters	492	14.44%
Genes assigned to COGs	2,502	73.42%
Genes assigned Pfam domains	2,623	76.97%
Genes with signal peptides	1,073	31.48%
Genes with transmembrane helices	812	23.83%
CRISPR repeats	1	

**Table 4:** Number of genes associated with the general COG functional categories

Code	value	%age	Description
J	155	5.6	Translation, ribosomal structure and biogenesis
A	1	0.1	RNA processing and modification
K	128	4.6	Transcription
L	154	5.6	Replication, recombination and repair
B	5	0.2	Chromatin structure and dynamics
D	28	1.0	Cell cycle control, cell division, chromosome partitioning
Y	0	0.0	Nuclear structure
V	45	1.6	Defense mechanisms
T	297	10.8	Signal transduction mechanisms
M	184	6.7	Cell wall/membrane/envelope biogenesis
N	106	3.8	Cell motility
Z	0	0.0	Cytoskeleton
W	0	0.0	Extracellular structures
U	83	3.0	Intracellular trafficking and secretion, and vesicular transport
O	106	3.8	Posttranslational modification, protein turnover, chaperones
C	274	9.9	Energy production and conversion
G	96	3.5	Carbohydrate transport and metabolism
E	185	6.7	Amino acid transport and metabolism
F	66	2.4	Nucleotide transport and metabolism
H	145	5.3	Coenzyme transport and metabolism
I	74	2.7	Lipid transport and metabolism
P	123	4.5	Inorganic ion transport and metabolism
Q	40	1.5	Secondary metabolites biosynthesis, transport and catabolism
R	274	9.9	General function prediction only
S	195	7.1	Function unknown
-	906	26.6	Not in COGs
